# Tumor biology, treatment patterns, and recurrence in breast cancer patients aged 70–79 vs ≥ 80 years: a large-scale registry analysis

**DOI:** 10.1007/s00404-026-08379-2

**Published:** 2026-03-28

**Authors:** F. Ganster, S. Schrodi, M. Braun, Ch. Seifert, S. Mahner, T. Kolben, R. Wuerstlein, N. Harbeck, S. Beyer, M. Burgmann

**Affiliations:** 1 Breast Center, Department of Gynecology and Obstetrics and Comprehensive Cancer Center Munich LMU, Bavarian Cancer Research Center (BZKF), LMU University Hospital, Marchioninistr. 15, 81377 Munich, Germany; 2 Bavarian Cancer Registry, Bavarian Health and Food Safety Authority (LGL), Munich, Germany; 3 Breast Center, Department of Gynecology, Red Cross Hospital, Munich, Germany

**Keywords:** Breast cancer, Tumor biology, Survival, Time trend, Elderly patients

## Abstract

**Purpose:**

With increasing life expectancy, breast cancer (BC) in elderly women is rising, yet patients over 80 remain underrepresented in clinical trials. Understanding differences in the age groups is essential to avoid over- and undertreatment. This analysis investigates age-specific tumor characteristics, treatment patterns and outcomes in women aged 70–79 versus ≥ 80 years.

**Methods:**

This population-based analysis included BC patients treated at LMU Breast Center and Munich Red Cross Hospital between 2004 and 2015. Clinical data of 2699 women aged 70–79 and those ≥ 80 were compared to assess differences in tumor biology, treatment approaches and time to metastasis.

**Results:**

Breast-conserving surgery rates remained stable over time, while sentinel lymph node biopsy use increased in both age groups. Patients aged ≥ 80 more frequently presented with larger tumors, underwent mastectomy, and received less systemic or axillary treatment, despite similarly high hormone-receptor positivity. Chemotherapy use peaked in 2009 and declined thereafter, whereas endocrine therapy (ET) increased steadily. Patients ≥ 80 received chemotherapy less often and more frequently ET alone. Across all biological subtypes, patients ≥ 80 were associated with higher rates of distant metastasis compared with those aged 70–79 years.

**Conclusion:**

Clinical and biological differences between women aged 70–79 and those ≥ 80 underscore the need for age-adapted, individualized BC management. Treatment decisions should carefully balance expected benefit against comorbidity burden and functional status. Refining age-specific treatment strategies may help to improve outcomes and quality of life, although this analysis cannot determine the effects of specific strategies in this growing patient population.

## What this analysis adds to clinical practice

**Table:** 

In elderly women with breast cancer, treatment planning should explicitly consider life expectancy, comorbidities, and functional status when balancing oncologic benefit and treatment burden.	
In patients aged ≥ 70 years, chemotherapy use has remained low or declined over time, whereas endocrine therapy has become the predominant systemic treatment.	

## Introduction

Breast cancer (BC) remains a major global health concern and continues to rise in incidence across all age groups and regions [1]. As life expectancy increases worldwide, the number of elderly women diagnosed with BC is growing substantially [2], making it essential to understand their specific clinical needs. Women aged 70–79 commonly present with comorbidities such as cardiovascular disease, diabetes, and osteoporosis, which influence treatment tolerability and therapeutic decision-making [3]. Systemic therapies, including chemotherapy and endocrine therapy, may cause increased toxicity in this age group, requiring a balanced evaluation of risks and benefits [4]. When diagnosed early, older women often have a good prognosis [5], and tumor growth may be slower than in younger patients [3], permitting more individualized or less aggressive therapeutic approaches [3, 6]. Nonetheless, potential undertreatment has been identified as an important factor associated with recurrence and breast cancer–related mortality in elderly women [7], often driven by concerns that chemotherapy or surgery could worsen comorbid conditions or functional status [8]. Because endocrine therapies are generally better tolerated than chemotherapy [9] and most tumors in elderly women are hormone-receptor positive [10], ET is frequently used in this population. Surgical decision-making also requires individualized assessment of oncologic benefit versus operative risk [11]. In addition to physical health, cognitive impairment and psychological burdens—such as anxiety or depression—may complicate treatment adherence and influence overall patient experience [12, 13]. Many older women prioritize maintaining quality of life, often selecting treatment options with fewer side effects [14, 15]. With the high prevalence of hormone receptor–positive disease in this age group [16], endocrine therapy often provides effective disease control [17]. Ultimately, comprehensive, individualized treatment planning is essential to avoid both over- and undertreatment [18].

Despite increasing recognition of these complexities, there remains limited evidence on how clinical features, treatment decisions, and outcomes differ within the elderly population itself—particularly between women aged 70–79 and those over 80, who differ markedly in comorbidity burden, physiological reserve, and life expectancy. This gap persists largely because very elderly women are underrepresented in clinical trials [3, 19, 20] and because most existing studies include small sample sizes or single-center retrospective cohorts [21–24]. Our analysis addresses this gap by analyzing a large, real-world cohort in two German breast centers, encompassing all women aged 70–79 and ≥ 80 treated at two major breast centers over an 11-year period. This extensive dataset provides a rare opportunity to examine age-specific differences in tumor biology, treatment patterns, and outcomes with far greater granularity and statistical robustness than previously available. The aim of this analysis is therefore to use this large cohort to identify meaningful distinctions between these two elderly age groups, assess whether treatment is appropriately individualized, and highlight opportunities to refine age-adapted care for an expanding population of older breast cancer patients.

## Methods

### Data collection

The data for this analysis were provided by the former Munich Cancer Registry (MCR) of the Tumor Center Munich (TZM), which served a population of about 5 million in Bavaria/Southern Germany. All pathology reports in the region were submitted to the registry, along with patient demographics, treatment details, and follow-up information. Vital status was updated using death certificates. The data was documented according to the International Agency for Research on Cancer guidelines. In 2018, data reporting responsibilities were transferred to the Bavarian Cancer Registry, now managed by the Bavarian State Office for Health and Food Safety (LGL).

### Cohort selection

Of the 11,954 invasive breast cancer patients diagnosed between 2004 and 2015 at either the LMU Breast Center or the Breast Center of Red Cross Hospital, 2699 elderly patients (aged 70–79 and ≥ 80 years) were included in the analysis. Exclusion criteria were made based on male sex, histology of lymphoma, sarcoma or non-invasive tumors and primary metastasis (M1). For the survival analysis, patients with a history of other previous or synchronous malignant tumors were also excluded to avoid any potential confounding effects.

### Definition of variables

Tumors were classified according to the TNM Classification of Malignant Tumors [25]. Since molecular subtypes were not available in the cancer registry, an alternative classification was used, based on estrogen receptor (ER), progesterone receptor (PR), HER2 expression, Ki-67, and tumor grade. Five subgroups were defined: "Luminal A-like" (HER2-, ER and/or PR + , Ki-67 < 10 or Grade 1/2); "Luminal B-like (HER2-)" (HER2-, ER and/or PR + , Ki-67 ≥ 10 or Grade 3); "Luminal B-like (HER2 +)" (HER2 + , ER and/or PR +); "HER2-like non-luminal" (HER2 + , ER-, PR-), and "Triple negative" (HER2-, ER-, PR-). ER and PR were considered positive if at least 1% of cells were positive. HER2 expression was evaluated using immunohistochemistry (IHC) and in situ hybridization (FISH/chromogenic in situ hybridization), following the ASCO/CAP guidelines [26].

### Statistical analysis

The MCR organized data in an Oracle database, and statistical analyses were performed using SAS (version 9.4; SAS Institute, Cary, NC). Prognostic factors and therapeutic interventions were analyzed by means of descriptive statistics. For the survival analysis, overall survival (OS) and relative survival (RS) were calculated by applying the Kaplan–Meier method. The expected survival time for age-matched individuals was obtained by using life tables for the German population and applying the Ederer II method [27]. RS represents relative survival from cancer after adjusting for other causes of death, and as such, it was used as an estimate of cancer-specific survival. Additionally, time to local recurrence (TTLR) and time to metastasis (TTM) were included as endpoints in the analysis, serving as surrogate markers for overall survival.

## Results

### Descriptive statistics

Between 2004 and 2015, a total of 11,954 patients were diagnosed and treated for invasive breast cancer at the LMU Breast Center and the Munich Red Cross Hospital, qualifying them for inclusion in this analysis. Within this cohort, 1981 patients, representing 16.6%, were aged between 70 and 79 years, 718 patients were 80 years or older and fulfilled the specified inclusion criteria.

The most prevalent diagnosis in patients aged 70–79 years was breast cancer at stage pT1, which constituted 1085 cases (60.65%). This was followed by 579 patients with stage pT2 (32.36%), 87 with stage pT3 (4.86%), and 38 with stage pT4 (2.12%). In the ≥ 80 group, stage distribution shifted slightly: 271 cases were pT1 (44.28%), 258 pT2 (42.16%), 31 pT3 (5.07%), and 52 pT4 (8.50%).

When examining nodal status, more than half of the 70–79 group were classified as pN0 (n = 1223; 64.06%), followed by pN + (n = 611; 32.01%) and pNx (n = 75; 3.93%). In contrast, the ≥ 80 group showed lower rates of node-negative disease: pN0 (n = 240; 39.09%), pN + (n = 207; 33.71%), and pNx (n = 167; 27.20%).

Tumor grading in the 70–79 cohort was predominantly Grade 2 (n = 1236; 63.03%), followed by Grade 3 (n = 424; 21.62%) and Grade 1 (n = 301; 15.35%). Similarly, in the ≥ 80 cohort, Grade 2 was also most common (n = 451; 63.61%), with Grade 3 (n = 170; 23.98%) and Grade 1 (n = 88; 12.41%) less frequent.

Regarding receptor status, patients aged 70–79 years had high rates of hormone receptor positivity: ER-positive (n = 1761; 89.25%), PR-positive (n = 1581; 80.17%), and combined ER/PR positivity in 90.22%. Among ≥ 80-year-olds, similar distributions were observed: ER-positive (n = 638; 89.61%), PR-positive (n = 571; 80.20%), and ER/PR positivity in 91.15%.

HER2 status was negative in the majority of cases in both groups: 88.76% (n = 1738) in the 70–79 cohort and 87.61% (n = 615) in the ≥ 80 cohort. HER2 positivity was 8.89% (n = 174) in 70–79-year-olds and 8.83% (n = 62) in those ≥ 80.

The most frequent biological subtype in both age groups was Luminal A-like, comprising 54.04% (n = 1031) in patients aged 70–79 and 55.19% (n = 372) in patients ≥ 80. Luminal B-like (HER2–) tumors accounted for 29.87% (n = 570) in the younger elderly and 28.78% (n = 194) in the oldest cohort. Luminal B-like (HER2 +) subtypes were observed in 6.24% (n = 119) of 70–79-year-olds and 7.27% (n = 49) of ≥ 80-year-olds. HER2 + /non-luminal tumors were diagnosed in 2.88% (n = 55) of the younger cohort and 1.93% (n = 13) of the ≥ 80 cohort, while triple-negative breast cancer accounted for 6.97% (n = 133) and 6.82% (n = 46), respectively.

An overview of the descriptive statistics for both cohorts is provided in Table 1

**Table 1:** Tumor classification, tumor biology and treatment of breast cancer patients 70–79 vs > 80 years

Tumor classification	70–70 years	80 years
Tumor size		
T1	n = 1085, 60.65%	n = 271, 44.28%
T2	n = 579, 32.36%	n = 258, 42.16%
T3	n = 87, 4.86%	n = 31, 5.07%
T4	n = 38, 2.12%	n = 52, 8.50%
Nodal status		
N0	n = 1223, 64.06%	n = 240, 39.09%
N +	n = 611, 32.01%	n = 207, 33.71%
Nx	n = 75, 3.93%	n = 167, 27.20%
Grading		
G1	n = 301, 15.35%	n = 88, 12.41%
G2	n = 1236, 63.03%	n = 451, 63.61%
G3	n = 424, 21.62%	n = 170, 23.98%
Tumor biology		
ER		
Positive	n = 1761, 89.25%	n = 638, 89.61%
Negative	n = 212, 10.75%	n = 74, 10.39%
PR		
Positive	n = 1581, 80.17%	n = 571, 80.20%
Negative	n = 391, 19.83%	n = 141, 19.80%
ER/PR		
Positive	n = 1780, 90.22%	n = 649, 91.15%
Negative	n = 193, 9.78%	n = 63, 8.85%
Her2-status		
Positive	n = 174, 8.89%	n = 62, 8.83%
Negative	n = 1738, 88.76%	n = 615, 87.61%
Her2 (2 +)	n = 46, 2,35%	n = 25, 3.56%
Subtype		
Luminal-A like	n = 1031, 54.04%	n = 372, 55.19%
Luminal-B like (Her2-)	n = 570, 29.87%	n = 194, 28.78%
Luminal-B like (Her2 +)	n = 119, 6.24%	n = 49, 7.27%
Her2 + /non-luminal	n = 55, 2.88%	n = 13, 1.93%
Triple negative	n = 133, 6.97%	n = 46, 6.82%
Local therapy		
Breast surgery		
No surgery	n = 31, 1.60%	n = 33, 5.05%
Breast conserving surgery	n = 1433, 73.90%	n = 370, 56.57%
Mastectomy	n = 475, 24.50%	n = 251, 38.38%
Axillary surgery		
No axillary surgery	n = 142, 7.17%	n = 268, 37.33%
LAD	n = 283, 14.29%	n = 106, 14.76%
SLNB + LAD	n = 314, 15.58%	n = 60, 8.36%
SLNB only	n = 1152, 58.15%	n = 205, 28.55%
Other axillary surgery	n = 90, 4.54%	n = 76, 11.00%
Radiation		
Yes	n = 1162, 81.09%	n = 179, 48.38%
No	n = 271, 18.91%	n = 191, 51.62%
Radiation after mastectomy		
Yes	n = 161, 33.89%	n = 48, 19.12%
No	n = 314, 66.11%	n = 203, 80.88%
Systemic Therapy**		
Chemotherapy, endocrine therapy		
No systemic therapy	n = 365, 18.43%	n = 207, 28.83%
Chemotherapy only	n = 106, 5.34%	n = 9, 1.25%
Endocrine therapy only	n = 1312, 66.23%	n = 496, 69.08%
Chemotherapy and endocrine therapy	n = 198, 9.99%	n = 6, 0.84%
Targeted therapy		
Yes	n = 99, 56.90%	n = 19, 30.65%
No	n = 75, 43.10%	n = 43, 69.35%

*ER* Estrogen receptor, *PR* Progesterone receptor, *LAD* Locally axillary dissection, *SLNB* Sentinel lymph node biopsy

^*^Her2 positive: IHC Score = 3 or IHC Score = 2 and FISH-Test positive. Her2 negative: IHC Score = 1 or IHC Score = 2 and FISH-Test negative

^**^Therapy Yes: recommended, started, completed. Therapy No: contraindicated, rejected by the patient, not completed

**Fig. 1:**
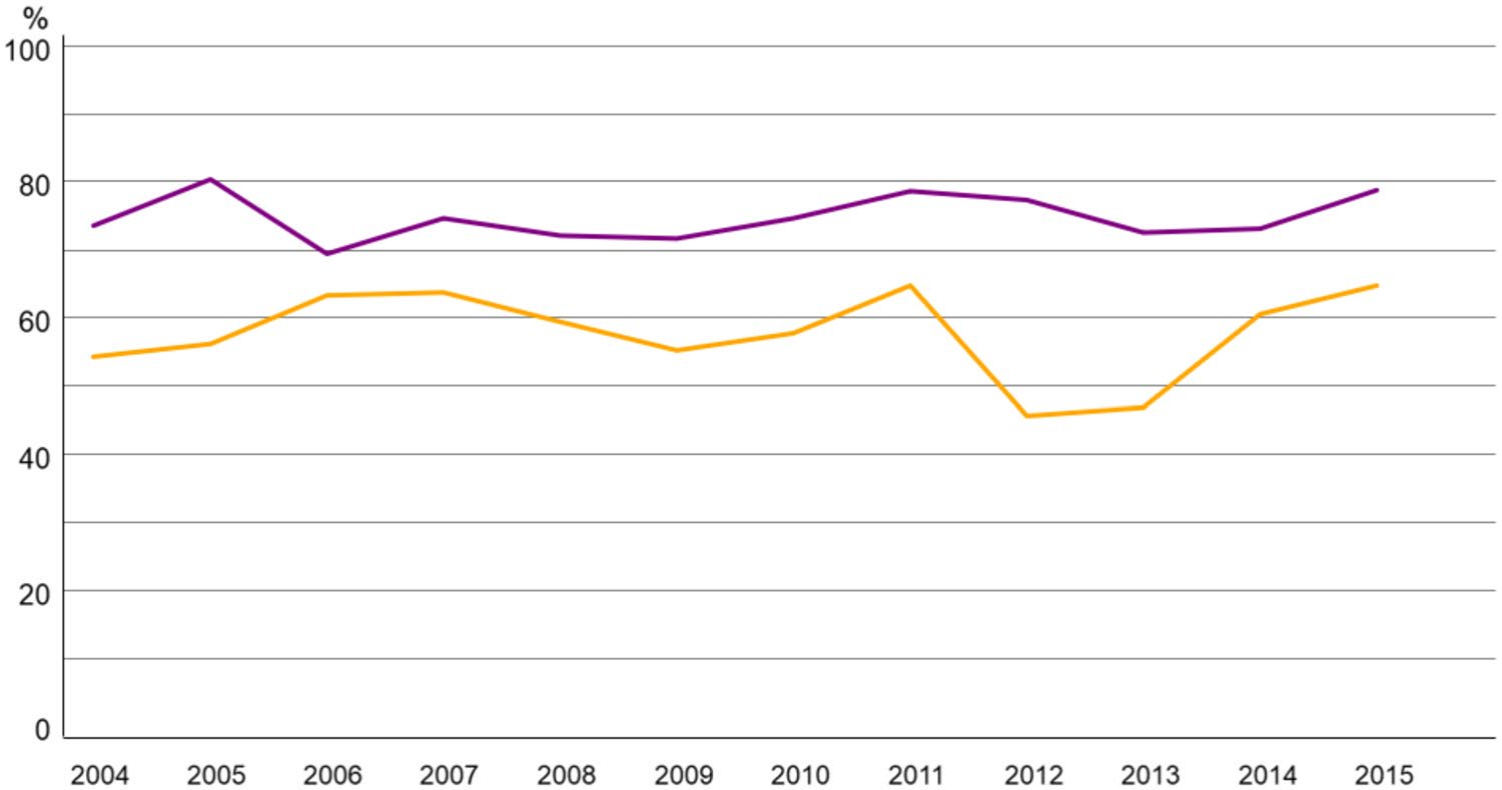
Trend of Breast-conserving surgery in breast cancer patients 70–79 (purple) vs > 80 years (yellow)

Most of the patients underwent breast-conserving surgery (BCS) (Fig. 1). The rates remained stable from 2004 to 2015 in both age groups, with a slight increase up to 2011 followed by a decline among patients ≥ 80 and a renewed increase afterward.

**Fig. 2:**
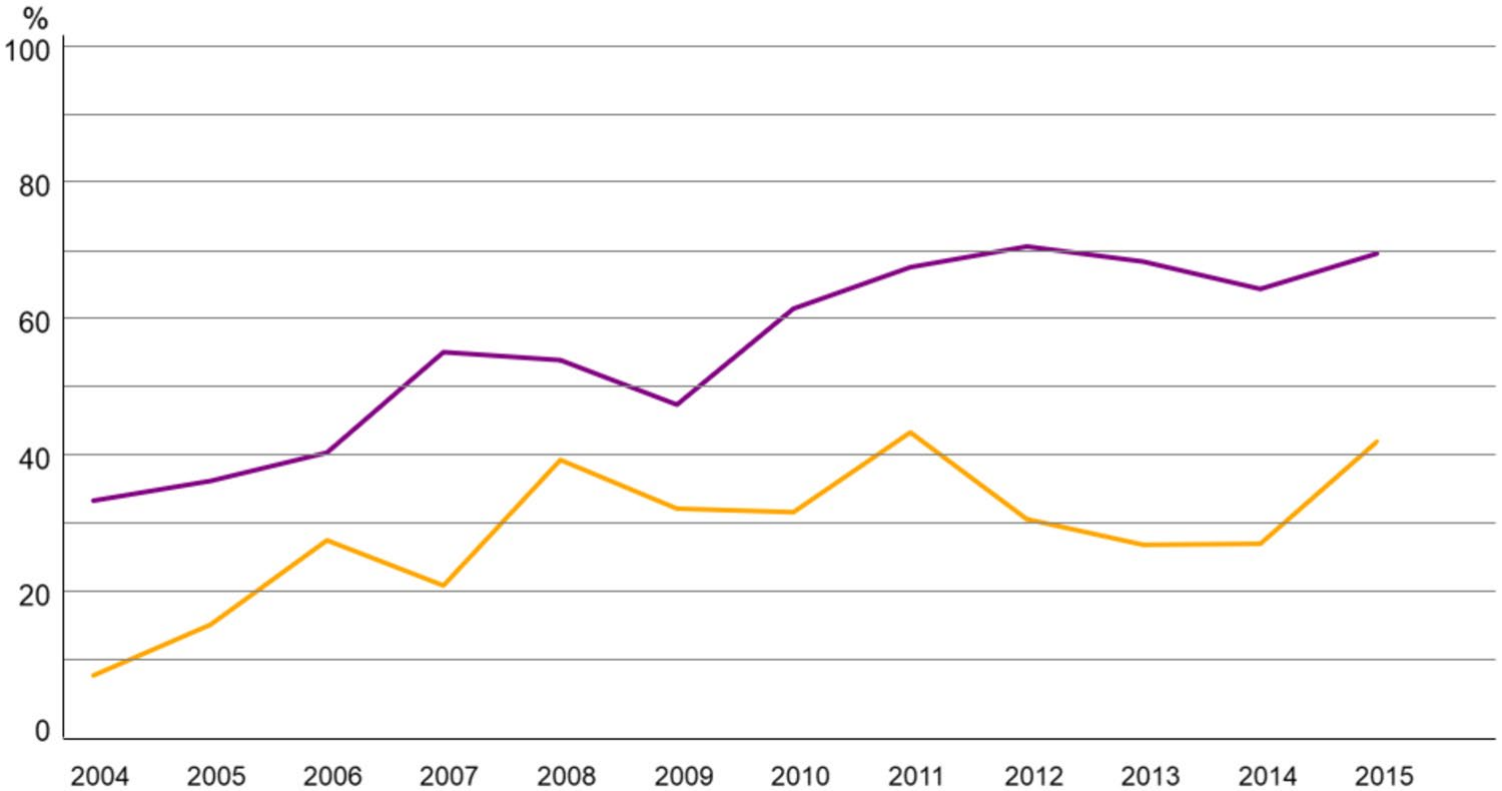
Trend of Sentinel lymph node biopsy (SLNB) alone in breast cancer patients 70–79 (purple) vs > 80 years (yellow)

Regarding axillary surgery, most patients in both age groups underwent sentinel lymph node biopsy (SLNB) alone (n = 1152, 58.2% in 70–79; n = 205, 28.6% in ≥ 80). As illustrated in Fig. 2, SLNB use rose steadily over time in both groups, stabilizing at 60–70% in the 70–79 cohort, whereas uptake was lower in patients ≥ 80.

**Fig. 3:**
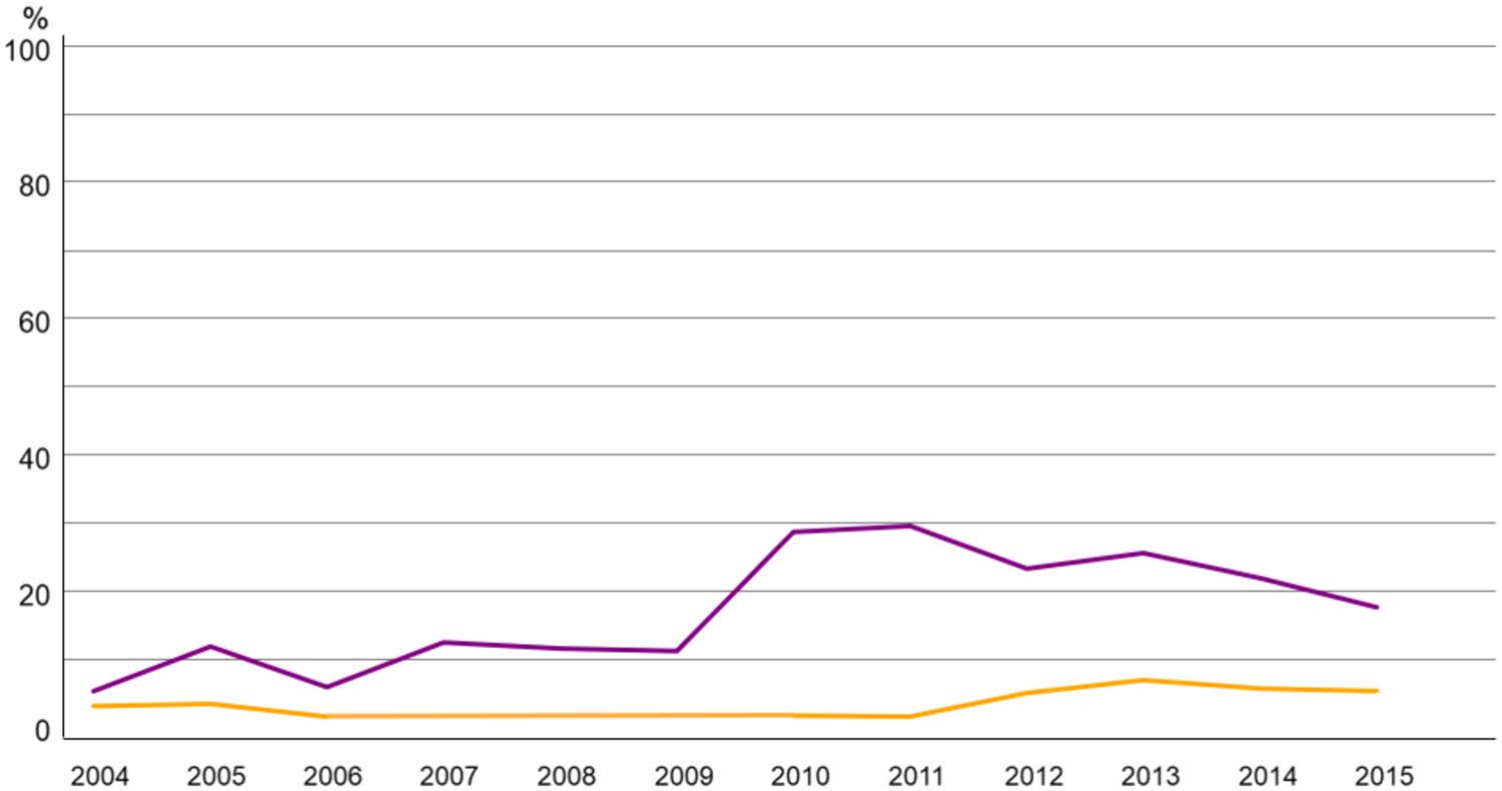
Trend of Chemotherapy alone in breast cancer patients 70–79 (purple) vs > 80 years (yellow)

**Fig. 4:**
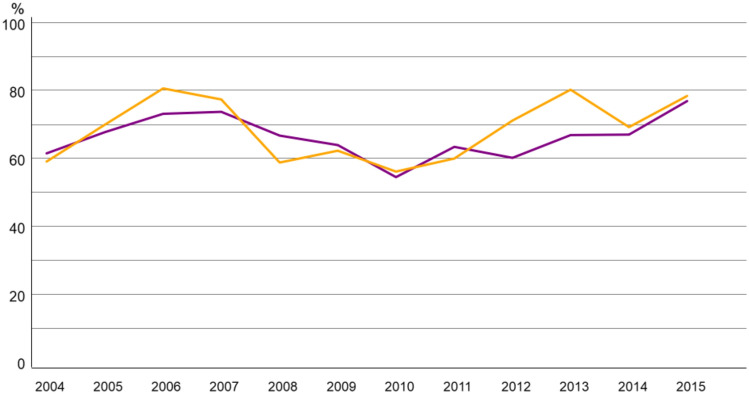
Trend of Endocrine therapy alone in HR positive breast cancer patients 70–79 (purple) vs > 80 years (yellow)

For systemic therapy, chemotherapy use was low in both age groups (5.3% in 70–79; 1.3% in ≥ 80), showing a temporary increase after 2009 before declining again (Fig. 3). Endocrine therapy was the most frequently applied systemic treatment, with a clear upward trajectory in both cohorts during the analysed period (Fig. 4).

### Local recurrence and metastasis

**Fig. 5:**
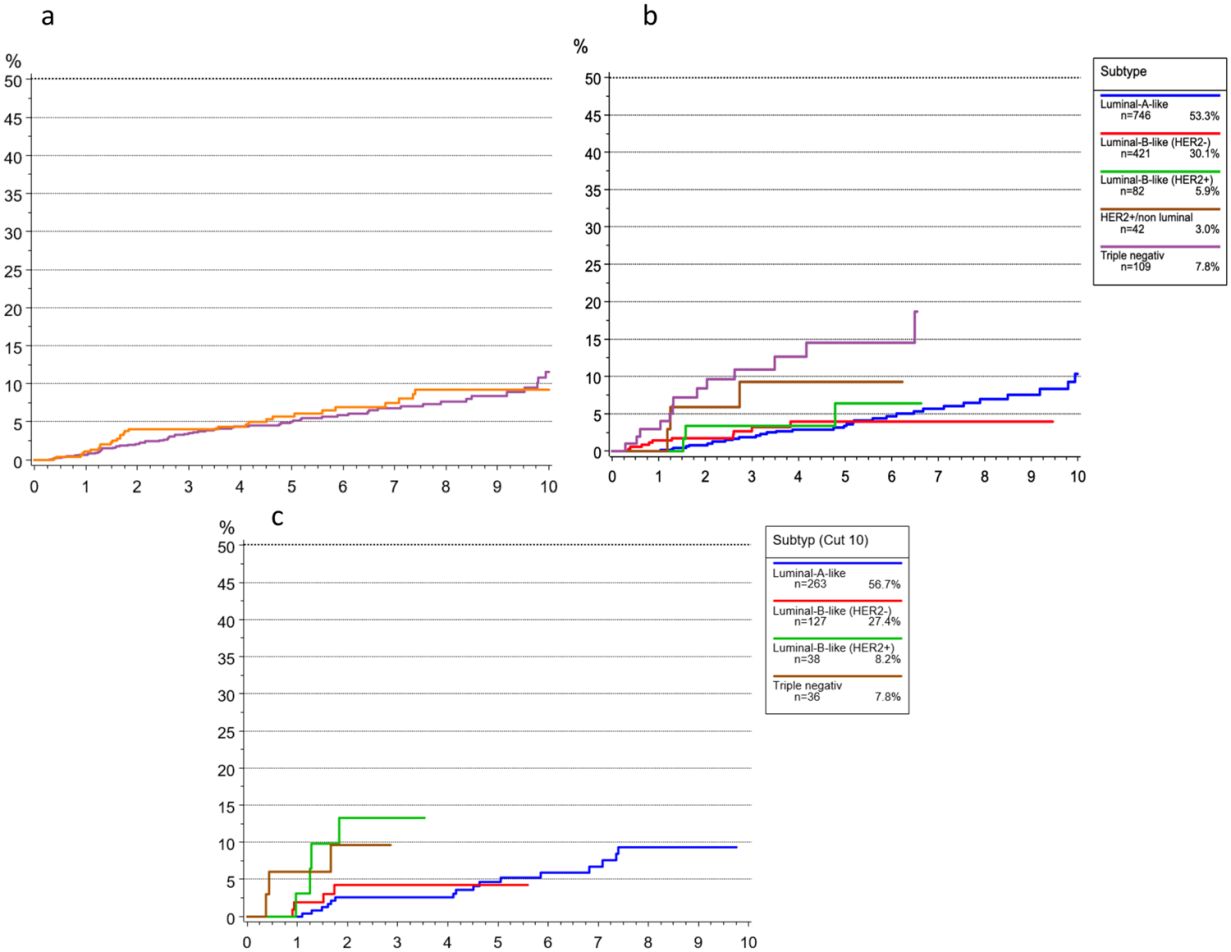
Cumulative incidence for time to local recurrence (TTLR) breast cancer patients **a** 70–79 vs > 80 years **b** Subtype 70–79 and **c** Subtype > 80 years

The cumulative incidence of time to local recurrence (TTLR) in the total elderly cohort was 5.0% after 5 years and 11.5% after 10 years (Fig. 5). Patients aged 70–79 years showed lower and more delayed recurrence rates compared with those ≥ 80 years, who experienced earlier and more frequent local relapses. Across both age groups, the highest recurrence rates were observed in patients with triple-negative breast cancer (TNBC) and HER2 + /non-luminal tumors, whereas Luminal A-like and Luminal B-like subtypes demonstrated the lowest and most delayed recurrence patterns.

**Fig. 6:**
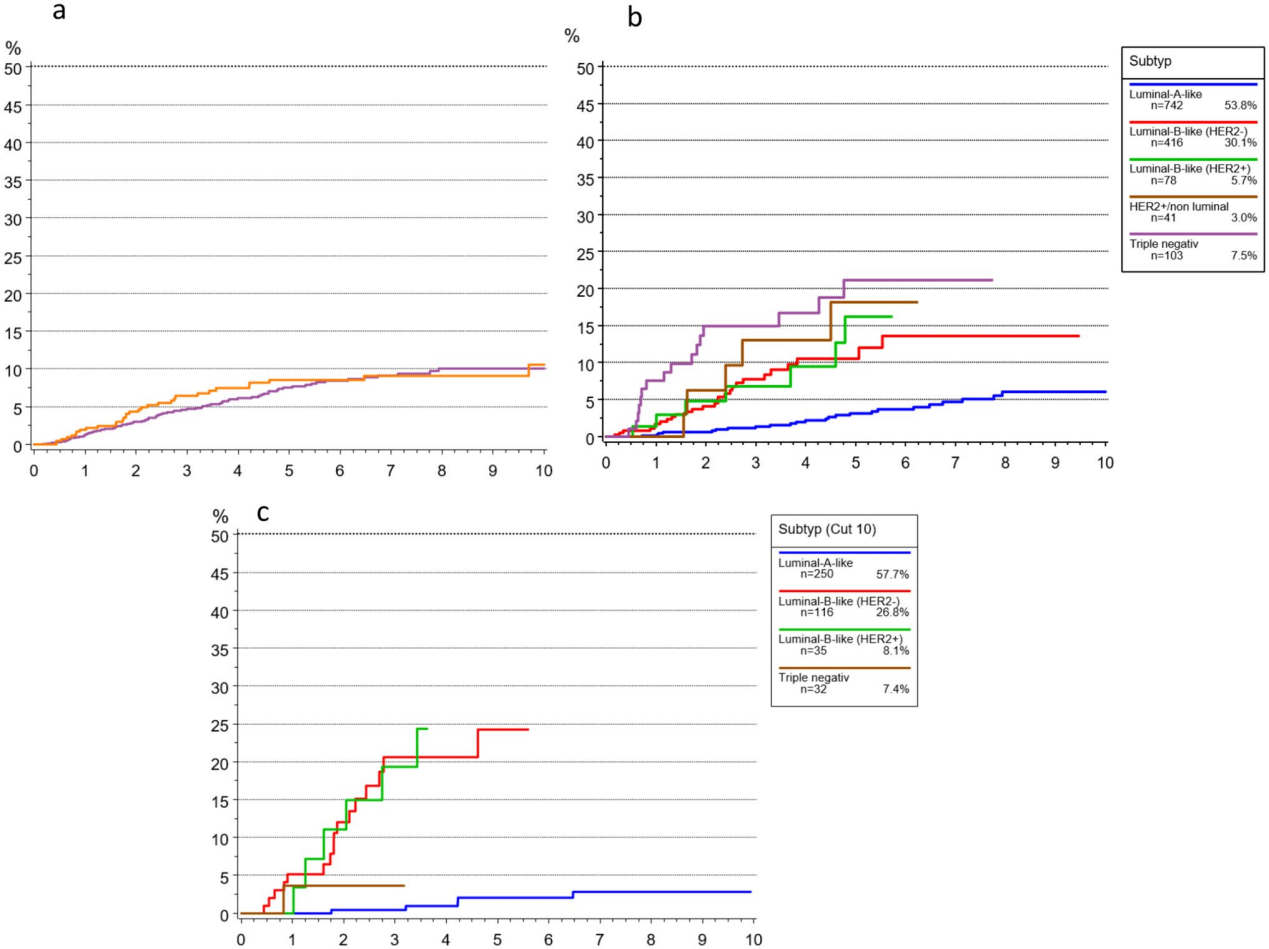
Cumulative Incidence of time to distant metastasis (TTM) according to subtype for breast cancer patients **a** 70–79 vs > 80 years **b** Subtype 70–79 and **c** Subtype > 80 years

The cumulative incidence of time to distant metastasis (TTM) for all elderly patients was 7.5% after 5 years and 10% after 10 years (Fig. 6). TNBC and HER2 + /non-luminal tumors carried the highest risk of metastasis, with rates approaching 20% after 5 years, while Luminal A-like cancers had the most favorable outcomes, with only ~ 3% developing distant metastases within 5 years. Patients aged ≥ 80 years consistently showed higher observed rates of distant metastasis compared with those aged 70–79 years, across all biological subtypes.

## Discussion

By means of this large-scale analysis we investigated breast cancer trends in patients aged 70 to 79 years and those ≥ 80 years treated at the LMU Breast Center and Munich Red Cross Hospital from 2004 to 2015. We described prognostic factors, treatment modalities, and clinical outcomes across both elderly cohorts. In the 70–79 group, more than 60% of the patients were diagnosed with stage T1 and Grade 2 tumors. In contrast, patients ≥ 80 years more frequently presented with larger tumor sizes (higher rates of pT2–pT4) and a greater proportion of unknown nodal status (pNx). These differences may partly reflect disparities in early detection: in Germany, the organized mammography screening program has historically included women aged 50–69 years, resulting in limited routine imaging for women ≥ 70[28]. Consequently, older patients may be less likely to undergo regular breast imaging and more likely to present with clinically detected or advanced-stage tumors [3]. Nonetheless, hormone-receptor positivity was very high in both groups, exceeding 89%, and HER2 negativity was observed in nearly 90% [29].

As expected, the analysis indicates that the rate of breast-conserving surgery (BCS) remained stable over the 11-year period, but while it was the predominant approach in 70–79-year-olds, mastectomy was more common in patients ≥ 80. The rate of Sentinel Lymph Node Biopsy (SLNB) steadily increased in both groups, particularly after its introduction into the German guideline in 2008 [30], though uptake remained lower in ≥ 80-year-olds, where omission of axillary surgery was more frequent [31]. This pattern is consistent with recent Choosing Wisely SSO–ASCO recommendations supporting the omission of SLNB in carefully selected older patients with clinically node-negative, hormone receptor–positive, early-stage breast cancer [32]. However, given that many women ≥ 80 in Germany are diagnosed outside organized screening programs, resulting in more advanced disease or unknown nodal status, the generalizability of SLNB omission needs to be carefully individualized [33].

Chemotherapy (CT), at a low level before 2009, saw a sharp rise in 2010. This may be related to the change of the Breast Cancer Guidelines in 2009, which placed more emphasis on targeted and individualized tumor therapy: the molecular subtype was given greater consideration, genetic tests were introduced to calculate the individual risk of relapse (e.g. Oncotype DX [34]), and neoadjuvant chemotherapy was given greater importance [30]. However, the absolute proportion of patients receiving CT differed markedly between the two age groups: while 15% of patients aged 70–79 received chemotherapy, only about 2% of those ≥ 80 did. The subsequent decrease in CT use in both groups may reflect a shift towards more individualized treatment strategies, with recognition of the potential risks of chemotherapy in older women [3, 35]. This trend may be due to growing awareness of the increased vulnerability of elderly patients to chemotherapy-related toxicities, particularly in those with comorbidities [36, 37]. At the same time, the possibility of undertreatment in patients aged ≥ 80 years remains a critical concern, as withholding chemotherapy solely due to chronological age may compromise outcomes [7].

Endocrine therapy (ET) experienced a consistent increase in both age groups. In the 70–79 cohort, ET was applied in about two-thirds of patients, while in ≥ 80-year-olds almost 70% were treated with ET as monotherapy. This likely reflects its favorable tolerability and the predominance of hormone-receptor–positive tumors in elderly women. The development of novel ET regimens targeting pathways such as mTOR and CDK4/6 offers opportunities to further tailor endocrine-based treatment for both groups [38].

Time to metastasis (TTM) was shortest in TNBC and longest in Luminal A-like disease, regardless of age group. These findings suggest that while increasing age strongly influences OS, tumor biology remains a decisive prognostic factor across both elderly populations.

The trends observed in this analysis appear to reflect broader shifts in clinical practice and the evolving understanding of breast cancer treatment in older populations [39]. One of the most notable trends is the increasing use of SLNB, which aligns with a broader move towards less invasive surgical approaches in older patients [40, 41]. However, the lower uptake of SLNB in the ≥ 80 cohort also reflects clinical caution driven by frailty, multimorbidity, and limited life expectancy. Importantly, competing-risk analyses from large population-based studies have shown that in women ≥ 75 with multiple comorbidities, 10-year mortality from non–breast-cancer causes far exceeds breast cancer–specific mortality. Such data highlight that treatment decisions in the very elderly must balance oncologic benefit with the high likelihood of non-cancer–related mortality.

The stable rate of BCS suggests that this technique remains a preferred option in younger elderly patients with early-stage disease, while in ≥ 80-year-olds, more conservative surgical management was common, reflecting both comorbidity profiles and treatment priorities. The observed differences in survival outcomes among the molecular subtypes of breast cancer are in line with existing literature, with Luminal A-like breast cancers showing the best prognosis due to their lower aggressiveness and favorable response to endocrine therapy [42]. In contrast, the aggressive nature of triple-negative breast cancer, which is associated with poor prognosis and rapid metastasis, presents significant challenges in the management of both age groups [43]. Given the rapid progression of TNBC, more intensive and tailored therapeutic interventions may be considered in carefully selected subsets of patients [44–46], while acknowledging the limited evidence in very old and frail populations.

Elderly patients with breast cancer often present a range of comorbidities, which complicate treatment decisions and outcomes [47]. These comorbidities are particularly prevalent in patients ≥ 80, who frequently have multiple chronic conditions and functional limitations, influencing both therapy choices and survival [48, 49]. The challenges in treating older breast cancer patients therefore extend beyond biological and clinical aspects of the disease [14]. Physical limitations can impact on the effectiveness and tolerability of treatments such as chemotherapy or radiation [50, 51], and social or functional limitations may hinder treatment adherence [19]. Therefore, a holistic approach to care, including geriatric assessment, psychosocial support, and individualized treatment plans, is essential for optimizing outcomes, particularly in those ≥ 80 years.

In conclusion, the treatment of breast cancer in women aged 70–79 years and those ≥ 80 years presents unique challenges that require a multifaceted approach. While the younger elderly more often undergo guideline-based therapies including BCS, SLNB, and systemic treatment, Patients ≥ 80 frequently receive more conservative management, likely reflecting frailty and comorbidities, while also highlighting the ongoing challenge of distinguishing appropriate de-escalation from potential undertreatment. The integration of Choosing Wisely SSO–ASCO recommendations regarding SLNB omission emphasizes the need for individualized decision-making, particularly in populations with limited screening exposure and substantial comorbidity-related competing risks. As the population of elderly breast cancer patients continues to grow, it will be essential to further refine treatment strategies, integrate supportive care, and address the complex medical and psychosocial needs of both 70–79-year-olds and ≥ 80-year-olds in order to support survival outcomes and quality of life, while avoiding undertreatment.

### Limitations of the analysis

This retrospective, registry-based analysis from two breast centers is subject to residual confounding and does not allow causal inferences about the impact of specific treatments on outcomes. Detailed information on comorbidities, frailty, functional status, and formal geriatric assessment was not available, limiting our ability to distinguish appropriate, patient-centered de-escalation from potential undertreatment, particularly in women aged ≥ 80 years. Radiotherapy, endocrine therapy, chemotherapy, and targeted therapies may be underestimated because of incomplete documentation in the cancer registry, which could affect absolute treatment rates and time-trend estimates. Furthermore, we lack data on the underlying reasons for individual treatment decisions (e.g. patient preference, contraindications, or logistical factors), so interpretation of decision-making processes is restricted. Finally, the cohort reflects patients treated between 2004 and 2015 in two German centers, which may limit the generalizability of our findings to other healthcare systems and to more contemporary treatment standards.

## Data Availability

The datasets generated are available from the corresponding author on reasonable request.

## Declarations

### Content to participate

As this is an observational analysis, no informed consent was required.
